# Nanoemulsion‐based transdermal delivery of third‐generation steroidal and non‐steroidal aromatase inhibitors in preclinical models

**DOI:** 10.1111/cpr.13753

**Published:** 2024-09-29

**Authors:** Lanyang Gao, Lin Gao, Shiyao Huang, Lei Sun, Mei Li, Chen Shen, Youyou Chen, Ruihao Tan, Yuji Chen, Chengguo Zhan, Frank Heinrich Wieland, Yingying Liu, Yinan Zhang, Yao Luo

**Affiliations:** ^1^ Metabolic Hepatobiliary and Pancreatic Diseases Key Laboratory of Luzhou City, Academician (Expert) Workstation of Sichuan Province, Department of General Surgery (Hepatopancreatobiliary Surgery), Fundamental and Clinical Research on Mental Disorders Key Laboratory of Luzhou The Affiliated Hospital Southwest Medical University Luzhou China; ^2^ School of Chemical Science and Engineering Tongji University Shanghai China; ^3^ Department of Laboratory Medicine/Sichuan Medical Laboratory Clinical Medicine Research Center, Innovation Institute for Integration of Medicine and Engineering, West China Hospital Sichuan University Chengdu China; ^4^ School of Chemistry and Chemical Engineering, Frontiers Science Center for Transformative Molecules and National Center for Translational Medicine (Shanghai) Shanghai Jiao Tong University Shanghai China

## Abstract

Aromatase inhibitors are effective in treating hormone receptor‐positive breast cancer, particularly in postmenopausal women. However, the challenges of inconsistent dissolution, variable absorption and side effects with oral administration persist. To address these issues, transdermal delivery has emerged as a viable alternative. In our study, we have developed nanoemulsion‐based transdermal creams containing third‐generation aromatase inhibitors Exemestane (EXE) or Letrozole (LE) and evaluated their toxicity, anti‐tumour effects and androgenic potency using preclinical models including Bama minipigs, DMBA‐induced breast cancer rats and orchidectomized male rats. The results of our study are significant, suggesting that both creams effectively penetrated the skin, demonstrating an impressive anti‐breast cancer effect. Importantly, EXE cream had no organ toxicity at the tested dose, providing a reassuring safety profile for its use. In contrast, LE cream displayed reversible toxicity from drug molecule itself in animals at the given dose, dissipating after 3 weeks of withdrawal and recovery. This study establishes a solid foundation for the safe clinical use of third‐generation aromatase inhibitors. It highlights transdermal creams as a promising drug delivery carrier for administering them.

## Graphical abstract

The formulations of both steroidal and non‐steroidal aromatase inhibitors in transdermal creams have demonstrated the ability to permeate the skin and effectively impede breast cancer progression. In particular, steroidal aromatase inhibitor cream exhibited negligible animal toxicity and moderate androgenic activity. In contrast, non‐steroidal aromatase inhibitor cream displayed some reversible toxicity.

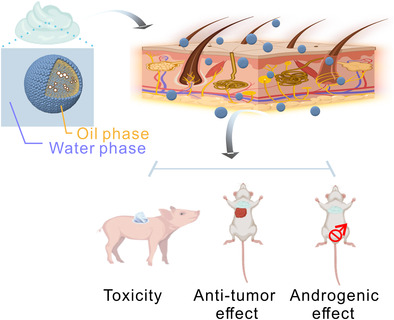

## 1 INTRODUCTION

The deprivation of oestrogen is widely recognized as the primary mechanism of action for medical and surgical endocrine treatments in breast cancer. Aromatase inhibitors (AIs), including steroidal Exemestane (EXE) and non‐steroidal Letrozole (LE), are effective in reducing oestrogen production in postmenopausal women.^1^, ^2^, ^3^, ^4^ These two drugs are potent aromatase inactivators with 25 and 2.5 mg tablets for oral administration, respectively.^5^, ^6^ However, taking these drugs orally poses challenges such as erratic dissolution, absorption and poor patient compliance with common adverse effects (body weight change, fatigue, dizziness, hot flushes, arthralgia and myalgias).^6^, ^7^, ^8^, ^9^, ^10^ The bioavailability of these drugs is limited by their suboptimal solubility and extensive first‐pass metabolism. Moreover, the absorption process of these drugs can vary significantly depending on factors including the formulation type and the concurrent food intake.^7^, ^11^

Transdermal delivery is often used as an alternative to oral administration to enhance the bioavailability of drugs.^12^, ^13^, ^14^ The skin offers a large surface area for drug absorption, making transdermal delivery appealing. It also bypasses first‐pass metabolism, reduces systemic toxicity and allows for the termination of drug action when needed.^15^ A topical formulation of aromatase inactivators (US 20030092693A1) was previously proposed to enhance the efficacy and convenience of drug administration. According to this patent, we prepared a Formestane cream and assessed its toxicity and anti‐tumour efficacy in animal models.^16^, ^17^ The results suggested that the cream suppressed breast cancer growth without inducing apparent damage to animal organs. However, as a second‐generation inhibitor, Formestane is less effective in inhibiting aromatase activity than the third‐generation inhibitors, EXE and LE.^18^

This study demonstrates nanoemulsion‐based transdermal delivery for steroidal and non‐steroidal third‐generation aromatase inhibitors EXE and LE with refined cream formulations. The toxicity of the creams was examined in female Bama minipigs treated for 28 days using a comprehensive analysis of haematology, biochemistry and histopathology. Furthermore, this study evaluated the anti‐tumour effects of the creams by monitoring the tumour size, tumour number and body weight of the dimethylbenz anthracene (DMBA)‐induced breast cancer rats after a four‐week treatment. The findings suggest that both creams effectively penetrated the skin and exerted a significant anti‐breast cancer effect. In particular, EXE cream demonstrated no toxicity on animal organs at the tested dose and showed the ability to activate androgen receptors, contributing to its anti‐breast cancer properties. On the other hand, the LE cream showed some toxicity in animals at the tested dose, which was reversible after a three‐week recovery. Our study paves the way to the clinical use of transdermal delivery of the third‐generation aromatase inhibitors.

## 2 MATERIALS AND METHODS

### 2.1 Structural characterization of the creams

The morphological and structural properties of EXE and LE creams were examined by cryo‐SEM (Apreo S, Aztec X‐Max80, AztecLi, ThermoFisher & Oxford Instruments) and transmission electron microscopy (JEM‐2100Plus, Jeol Ltd., Tokyo, Japan).

### 2.2 Toxicological evaluation

Sixteen minipigs were randomly divided into four groups (four pigs in each group). A cohort comprising four pigs per group was utilized for the safety evaluation. This decision aligned with the document ‘Technical Guidelines for Repeated Drug Administration Toxicity Testing’, ensuring adherence to relevant regulatory standards.^19^ The experimental groups were designated as follows: Normal (N), Placebo (P), Exemestane (EXE) and Letrozole (LE). Placebo means cream excipient.

1. Group N: Pigs in the normal group were not treated as the untreated, normal control group (N).
2. Group P: Animals in the placebo (P) group received transdermal administration of the cream excipient (1440 mg/kg bw/d) without any drug.
3. Group EXE: Each pig was administered a 1440 mg/kg bw/d dose of EXE cream, equivalent to 36 mg/kg bw/d of the active ingredient EXE.
4. Group LE: Each pig received a dose of 1440 mg/kg bw/d of LE cream, equivalent to 36 mg/kg bw/d of the active (LE).
5. Autopsy: Upon the conclusion of the experimental period on day 28, animals deemed to be in good condition underwent necropsy and macroscopic examination. However, those exhibiting poor conditions were allowed an additional 3‐week recovery period. Animals designated for dissection underwent anaesthesia with a dose of 35 mg/kg bw of 2% pentobarbital sodium, administered intramuscularly, followed by sacrifice through exsanguination. In the case where an animal was in a moribund state, rapid exsanguination was executed by severing the arteria crural, followed by a comprehensive autopsy. The procedure began with an external examination, opening the abdominal cavity. A macroscopic examination was then conducted by observing the appearance of tissues in situ. The cranial and thoracic cavities were opened and subjected to macroscopic examination. Any abnormalities detected during this process were meticulously recorded, including details of their location, colour, shape and size. All organs were systematically extracted from each animal, their weights recorded and then fixed in neutral buffered formaldehyde (4%) to facilitate subsequent histopathological examination.
6. Histopathological analysis: The collected organs were promptly immersed and fixed in 4% paraformaldehyde for 24 h to forestall any further alterations in the tissue. The tissue samples were carried out as reported.^17^ Fixed tissue sections were viewed under a binocular microscope at magnifications of 100 and 400 and photomicrographs were taken for further examination.

### 2.3 Evaluation of the anti‐breast cancer effect

The rats with DMBA‐induced breast tumours were divided into three groups, each consisting of 10 animals: the P group, the EXE group and the LE group. All animals were ovariectomized, and they represent postmenopausal breast cancer models.

Rats in the Placebo group received transdermal application of 125 mg/kg bw/d of placebo cream twice daily. Those in the EXE group were treated with EXE cream twice daily, administered transdermally at a dose of 125 mg/kg bw/d. The LE group received transdermal administration of 125 mg/kg bw/d of LE cream twice daily. All treatment regimens were upheld for 4 weeks. The selected doses for evaluating the anti‐tumour effect of the cream were determined based on prior studies conducted by Brodie.^20^ Tumour growth and the body weight of the rats were expressed as a percentage relative to the initial tumour volume, measured on the first day of treatment, and established as 100%. After the treatment period, euthanasia was conducted via cervical dislocation and the number of tumours was duly recorded.

### 2.4 Evaluation of the androgenic activities

The Hershberger assay was performed according to the guidelines of the rodent Hershberger assay^21^ to assess the androgenic and myotrophic activity of the creams. Male Wistar rats (140 g, 5 ~ 6 weeks) were ovariectomized (orchid) or sham‐operated (intact) under ether anaesthesia. After 14 days of endogenous hormonal decline, animals were randomly allocated to treatment and control groups (*n* = 6). The Orchi group refers to the Orchi rats receiving placebo cream; the Intact group refers to sham‐operated rats receiving placebo cream. The treatment group comprised orchid rats that were topically administered with 125 mg/kg bw/d of either EXE or LE cream. The anti‐androgen FLU given by gavage (10 mg/kg bw/d). The positive control group of rats received subcutaneous testosterone propionate (TP) administration at 1 mg/kg bw/d. Rats were sacrificed after completion of the 12‐day treatment. Following removal, the wet weights of the prostate, seminal vesicle and levator ani muscle were determined.

The animal experiments were performed under the Guideline for Care and Use of Experimental Animals and approved by the Institutional Review Board (or Ethics Committee) of The Affiliated Hospital of Southwest Medical University, Southwest Medical University (protocol code 201903‐37 and date of approval 5 March 2019).

### 2.5 Statistical analysis

Statistical analysis was performed utilizing the two‐tailed Student's *t*‐test and one‐way analysis of variance (ANOVA) via GraphPad Prism software (GraphPad Software). A *p*‐value less than 0.05 is considered statistically significant, while *ns* indicates non‐significance. Significance levels were denoted by asterisks (*p < 0.05, **p < 0.01, ***p < 0.001).

## 3 RESULTS AND DISCUSSION

### 3.1 Schematic illustration of the assessments of EXE and LE creams in animal models

**FIGURE 1:**
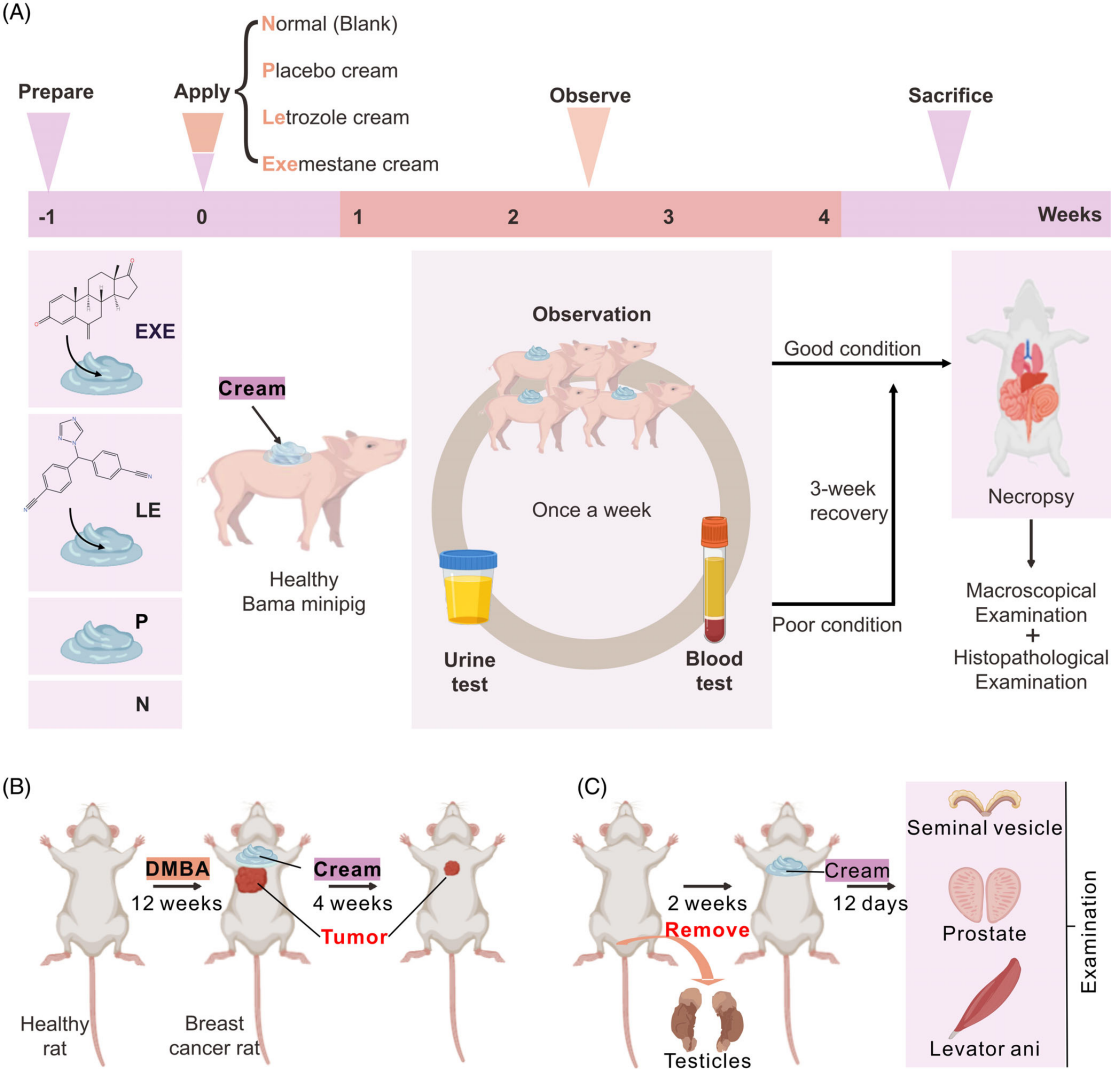
Schematic illustration of the assessments of EXE and LE creams in animal models. (A) Toxicological evaluation of the creams. (B) The effects of the creams on breast cancer. (C) Evaluation of androgen properties of the creams. N, Normal, pigs without any treatment. P, each pig received 1440 mg/kg bw/d of placebo cream. EXE, each pig received 1440 mg/kg bw/d of EXE cream. LE, each pig received 1440 mg/kg bw/d of LE cream.

We have developed a topical formulation based on the US patent document 20030092693A1 with some modifications. Specifically, we added dimethyl isosorbide to enhance the formulation's skin penetration and anticancer efficacy. We prepared the EXE or LE creams to determine if transdermal delivery is feasible for third‐generation aromatase inhibitors. The toxicity of these creams was examined in female Bama minipigs treated for 28 days. A comprehensive haematology, biochemistry and histopathology analysis was conducted to evaluate the effects (Figure 1A). At the end of the experimental period on day 28, necropsy and macroscopic examination were performed on animals in good condition. Those who were in poor conditions were allowed an additional 3‐week recovery period. To evaluate the anti‐cancer effectiveness of EXE or LE cream, rats are administered DMBA to induce tumours, followed by a four‐week treatment with either cream (Figure 1B). Additionally, previous studies have shown that steroidal aromatase inhibitors can act as androgens to inhibit breast cancer progression by activating androgen receptors.^22^ Therefore, we conducted a classical Hershberger assay to confirm the androgenic properties of the creams (Figure 1C).

### 3.2 Characterization of the creams

**FIGURE 2:**
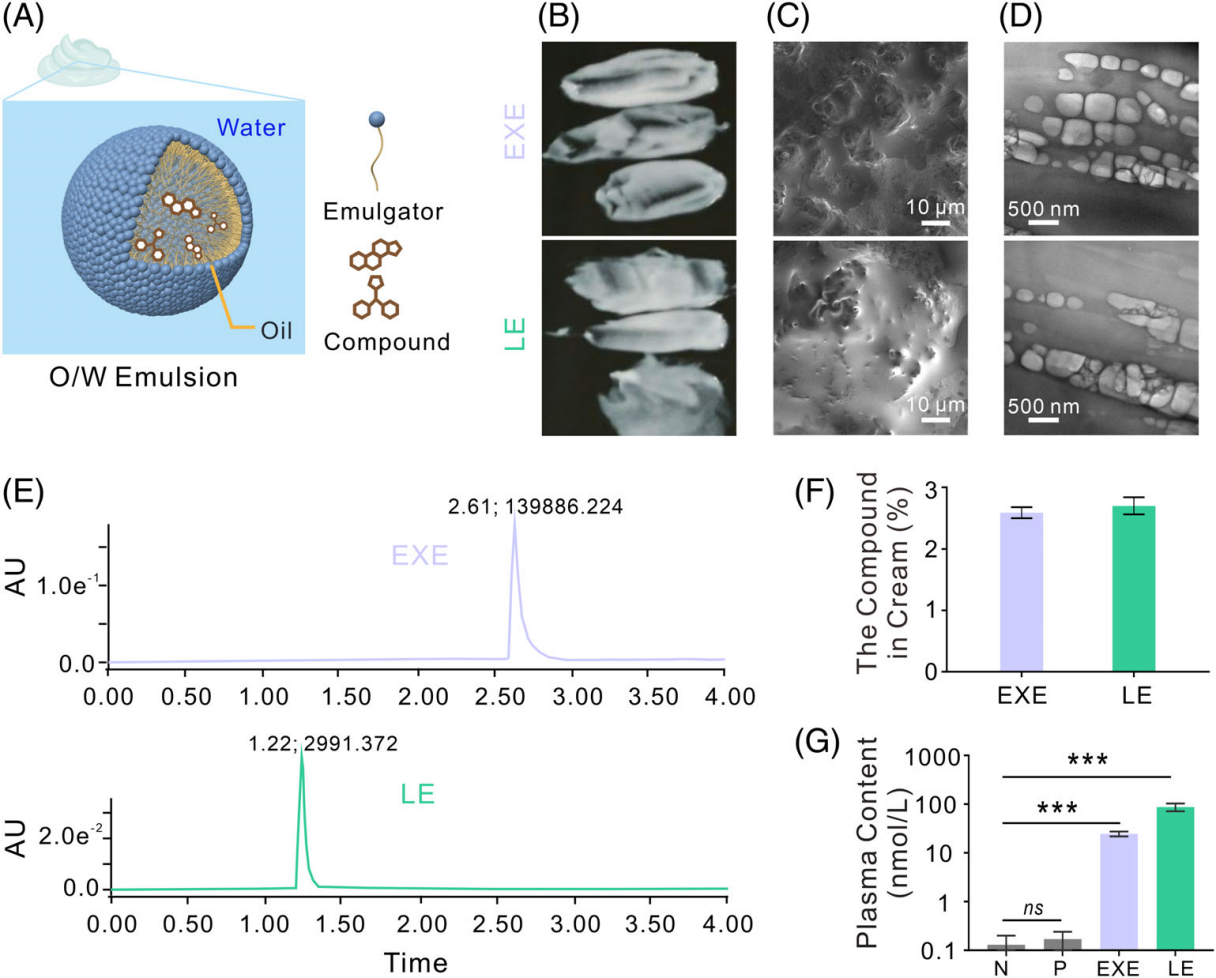
Characterization of EXE and LE creams. (A) Microstructural illustration of the creams. (B) Cream appearances. (C) Representative Cryo‐SEM images of the creams. Scale bars: 10 μm. (D) TEM images of the creams. Scale bars: 500 nm. In Figure b‐d, the upper images depict EXE cream, whereas the lower images represent LE cream. (E) UPLC analysis of LE and EXE content in creams. The peak of a single compound of EXE and LE. (F) LE and EXE content in creams. (G) LE and EXE plasma content in minipigs treated with LE or EXE cream for 2 weeks. The measurements were taken during the final 2 h of administration. N, Normal, pigs without any treatment. P, each pig received 1440 mg/kg bw/d of placebo cream. EXE, each pig received 1440 mg/kg bw/d of EXE cream. LE, each pig received 1440 mg/kg bw/d of LE cream.

The creams in this dosage form comprised an oil‐in‐water emulsion (Figure 2A). EXE and LE were solubilized in the oil phase, and then emulsification was used to create an even dispersion of tiny droplets in the water phase. The creams exhibited suitable consistency, good flexibility and coating properties and a smooth and uniform texture (Figure 2B). According to the cryo‐scanning electron microscopy (Cryo‐SEM) results, both creams have a smooth surface with holes (Figure 2C). The transmission electron microscopy (TEM) analysis revealed that they have oil droplets ranging from 200 to 300 nm in diameter (Figure 2D). These nanoscale droplets can enhance the permeability of the drug through the skin.^23^, ^24^, ^25^, ^26^ The concentration of the active ingredients in the cream was determined using UPLC assay. The analysis revealed that both creams contained a single compound, either EXE or LE (Figure 2E), and their respective concentrations were 2.59% and 2.70%. During a two‐week duration of treatment, the plasma concentrations of EXE and LE in pigs were measured as 24.64 and 87.56 nM, respectively (Figure 2F,G). These measurements were taken 2 h after administering the dose on day 7. These results demonstrate that both creams are nanoemulsions and can be absorbed through the skin of Bama minipigs and enter their bloodstream.

### 3.3 Effects of the creams on animal symptoms

During the study, female Bama minipigs were grouped and treated with the EXE, LE, and placebo (P) creams at a dose of 1440 mg/kg bw/d, respectively. The cream dose corresponds to about 36 mg/kg bw/d of active ingredients for Bama minipigs (see Supporting Material and Methods for details). Pigs without any treatment were assigned to the normal (N) group. They were closely monitored for any signs of toxicity or mortality, with observations being conducted twice daily to detect potential adverse effects promptly. No deaths were recorded during the study. However, after 3 weeks of medication, minipigs in the LE group exhibited signs of poor health, including thinness, lethargy, slow movement, decreased appetite, yellowing of the lips and dull eyes (Figure S1). Their body temperature remained normal, though the body weight and appetite of the pigs in this group decreased to some extent (Figure S2A,B and Table S1). The symptoms observed in the LE group were alleviated after discontinuing the drug, and the minipigs recovered after 3 weeks. This implies that the adverse effects associated with LE cream administration were reversible. The results also indicate that neither the administration of EXE cream nor the placebo cream induced any noticeable adverse effects within 3 weeks. Hence, the side effects of LE cream were caused by the LE compound itself rather than other cream components. The toxicity of LE cream to Bama minipigs at the administered dose of 1440 mg/kg bw/d is likely due to its relatively higher dose exceeding the clinical dose by more than 100 times (see Supporting Material and Methods for details). The commercially available oral formulations of EXE and LE were administered to patients at dose levels of 25 and 2.5 mg per application, respectively.^27^ The recommended clinical dosage of LE is 1/10 of that of EXE. This study has provided evidence of the safety of EXE cream at a dosage of 1440 mg/kg bw/d. Consequently, it can be inferred that the corresponding safe dosage of LE cream should be significantly lower, approximately 144 mg/kg bw/d. The safety profile of LE cream at the speculated dosage of 144 mg/kg bw/d requires additional validation through comprehensive animal studies.

### 3.4 Effects of the creams on animal haematology and clinical chemistry

Haematological parameters help assess the severity of the impact on the blood.^28^, ^29^, ^30^ The data reveals that EXE cream, administered at a dosage of 10 times higher than the clinical recommendation, did not cause any significant alterations in vital haematological parameters, including RBC, HGB, MCHC, PLT, WBC, LY and MID (Figure S2C–E and Table S2). However, the administration of LE cream at a dosage of 100 times higher than the clinical recommendation resulted in a sustained increase in HGB, WBC, LY and MID after the second week of dosing. These indicators did not exhibit a gradual decline until 2 weeks after the discontinuation of the drug. Despite higher HGB levels in the LE group compared to other groups (Figure S2C), the values remained within the normal range of HGB in Bama minipigs, eliminating concerns regarding abnormal elevation. However, further analysis is necessary to determine the underlying reasons for increased WBC, LY and MID levels in the LE cream group (Figure S2D). Previous research has reported cases of LE‐induced hepatitis with autoimmune characteristics.^31^ These findings are consistent with our observations of elevated WBC, LY and MID levels, collectively suggesting that LE triggered a drug‐induced inflammatory response. Regarding haematological parameters, the administration of EXE cream at the tested dose did not cause any adverse effects in the minipigs. In contrast, the dose of LE cream administered to the minipigs was excessive.

Any changes in the parameters of PT and APTT can serve as predictive markers of toxicity, especially when data from animal studies are extrapolated to human toxicity. Therefore, it is crucial to assess coagulation parameters to evaluate the risk of the tested substances on blood clotting mechanisms.^28^ The coagulation function of all animals was analysed both before and once a week after drug administration. It was found that both EXE and LE creams, administered at the investigated doses, did not induce significant changes in coagulation parameters (Table S3 and Figure S2F). These findings indicate that the two creams did not adversely affect the coagulation function of Bama minipigs.

The effect of creams on liver function was evaluated by assessing biochemical markers such as AST, ALT, GGT and other indicators. The results showed that EXE cream did not significantly affect the animals' liver function (Table S4 and Figure S3A–E). Blood biochemical parameters showed no significant differences between the normal and EXE groups. Furthermore, all groups' electrolyte indexes remained within the normal range (Table S3 and Figure S3F). In contrast, the LE group showed a continuous increase in TB, ALT and AST values after 14 days of dosing. These values significantly exceeded those in the normal control group after 21 days. Although these values gradually decreased after discontinuing the drug, they remained elevated compared to normal values 3 weeks after cessation (Tables S4,S5 and Figure S3D,E). This indicated jaundice symptoms and was consistent with a previously reported case where a patient developed jaundice after taking LE.^1^ Other blood biochemical values of minipigs in the LE group exhibited fluctuations within the normal range (Tables S4,S5 and Figure S3A–C). The elevated TB values in the LE group also explained the observed yellowing of the skin and lips in the pigs (Figure S1). There were no abnormal changes in erythrocytes and haemoglobin following LE application (Figure S2C,E). This suggests that LE did not induce hemolysis in Bama minipigs. The jaundice observed in the animals was attributed to impaired liver function rather than hemolysis. Therefore, the combination of jaundice and increased ALT and AST activities suggests that LE, at the tested dose, had a notable impact on the liver function of the animals within the LE group. In summary, the findings suggest that EXE cream was safe for the livers of Bama minipigs. However, the safety of LE cream for liver function in these animals warrants further investigation.

### 3.5 Effects of the creams on animal urine

After analysing the urine samples of female Bama minipigs, it was found that the use of EXE cream did not cause any significant changes in the levels of SG, pH, GLU, WBC, KET, BIL and URO when compared to the normal group or the placebo group (Table S6, Figure S2G). However, certain minipigs noted fluctuations in NIT, PRO and BLD levels. These changes were observed in the experimental and control groups, suggesting that they were unrelated to the drug but could be attributed to variations in urine collection methods or possible errors in the assay.

The use of LE cream in minipigs had a minor effect on the urinary levels of GLU, WBC, KET, SG and pH in the urine of minipigs. However, notable changes were observed in the NIT, PRO, BLD, BIL and URO levels. It is worth noting that alterations in NIT and PRO were also observed in the normal group, suggesting that these changes were likely unrelated to the drug and could be attributed to sampling and detection errors. The variation in BLD levels was only present after 7 days of drug administration and disappeared with the continuation of drug administration. This short‐lived abnormal alteration suggests that it was not related to the drug and might be influenced by other unknown factors. On the other hand, urinary BIL and URO began to show abnormal levels after 14 days of minipigs being topically applied with LE cream and persisted throughout the dosing period. These levels returned to normal after discontinuation of the drug (Table S7). This pattern strongly suggests that the compound LE caused the changes in BIL and URO. Abnormal levels of BIL and URO are typically associated with jaundice and liver injury. The supporting blood biochemical results have substantiated that LE induces liver injury and jaundice signs in pigs, explaining the abnormal levels of BIL and URO in the urine of minipigs in the LE cream group. These findings indicate that the application of EXE cream had no adverse effects on female Bama minipigs' kidney function. Conversely, the administered dose of LE cream impacted the kidney function of the minipigs.

### 3.6 Effects of the creams on animal organs

**FIGURE 3:**
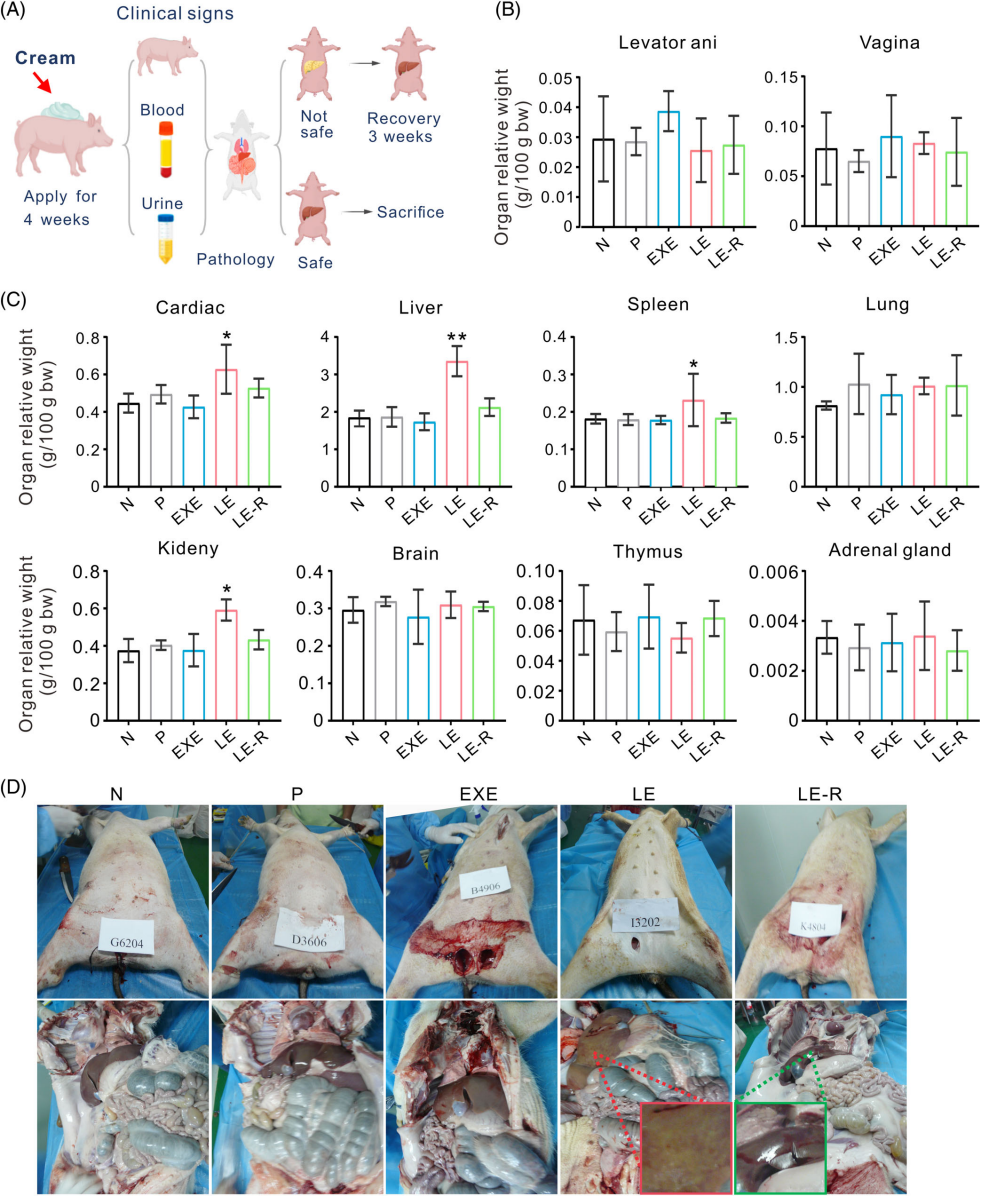
Toxicological evaluation of EXE and LE creams in Bama minipigs. (A) A scheme for assessing the toxicity of EXE and LE creams. (B, C) Relative organ weights of minipigs. (D) Gross anatomical photograph of minipigs. The upper images illustrate the general shape of pigs, while the lower images represent the state of their internal organs. The red box represents a typical liver with abnormalities. The green box represents a liver recovered 3 weeks after LE cream treatment. N, Normal, pigs without any treatment. P, each pig received 1440 mg/kg bw/d of placebo cream. EXE, each pig received 1440 mg/kg bw/d of EXE cream. LE, each pig received 1440 mg/kg bw/d of LE cream.

At the end of the study, female Bama minipigs were euthanized, and their main organs were examined for any gross abnormalities. The results revealed that no abnormalities in the EXE cream group were found in any of the examined organs, including the heart, liver, spleen, lung, kidney and cerebrum (Figures 3D and S4). In contrast, minipigs in the LE cream group showed emaciation, yellow skin coloration, liver swelling with a yellowish appearance, spleen enlargement, increased pulmonary nodules and other abnormal conditions. These pathological changes were reversible after discontinuing the drug (Figures 3D, S1, and S4). The results suggest that the tested dose of EXE cream had a negligible impact on the organ coefficients of minipigs (Figure 3A,C). Interestingly, an increase in levator ani was observed, which could be attributed to the androgenic effects of EXE (Figure 3B). On the other hand, LE cream adversely impacted the minipigs' internal organs. The organ coefficients of the heart, liver, spleen and kidney in LE‐treated minipigs were significantly higher than those in the normal group of minipigs (Figure 3C). These findings confirm that the repeated 28‐day administration of EXE cream did not cause any notable adverse effects on the internal organs of the animals. In contrast, LE cream caused damage to minipigs' organs, as evidenced by alterations in organ coefficients.

**FIGURE 4:**
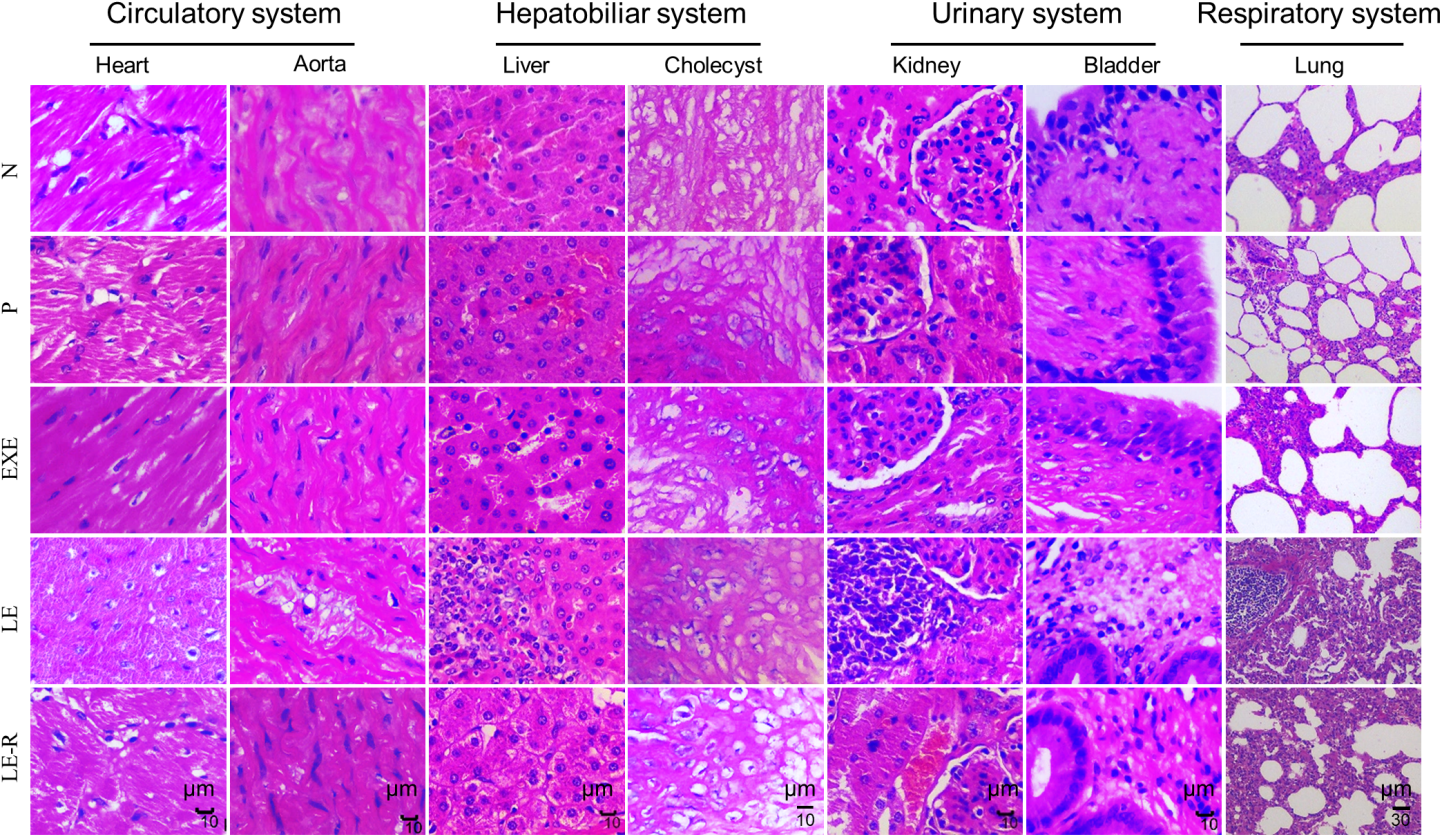
Histopathological examination of minipig organs. Sections observed at 400×, or 100× (lung) magnification. Histological structures were observed for the circulatory system (heart and aorta), hepatobiliary system (liver and cholecyst), urinary system (kidney and bladder), and respiratory system (lung). Scale bar: 10 or 30 μm (lung). N, Normal, pigs without any treatment. P, each pig received 1440 mg/kg bw/d of placebo cream. EXE, each pig received 1440 mg/kg bw/d of EXE cream. LE, each pig received 1440 mg/kg bw/d of LE cream. LE‐R, recovery continued for an additional 3 weeks after day 28 in the minipigs from the LE group.

Tissue sections demonstrate that the EXE cream primarily suppressed the development of mammary glands and caused minimal pathological impact in other tissues (Figures 4, S5, and S6). This is supposedly because EXE is an aromatase inhibitor and reduces oestrogen production.^32^ On the other hand, LE cream substantially damaged several organs in Bama minipigs, including the aorta, liver, kidney, bladder and lungs. However, the immune, nervous and endocrine systems remained unaffected (Figures 4, S5, and S6). Both LE and EXE cream exhibited inhibitory effects on the growth of mammary glands in female Bama minipigs (Figure S5). These findings indicate that EXE cream is non‐toxic at the tested dose. However, the LE cream, at a dose of 1440 mg/kg bw/d, exhibited toxic effects on pigs. Further investigation is necessary to determine a safe concentration for the topical application of LE cream.

### 3.7 The anti‐breast cancer effects of the creams

**FIGURE 5:**
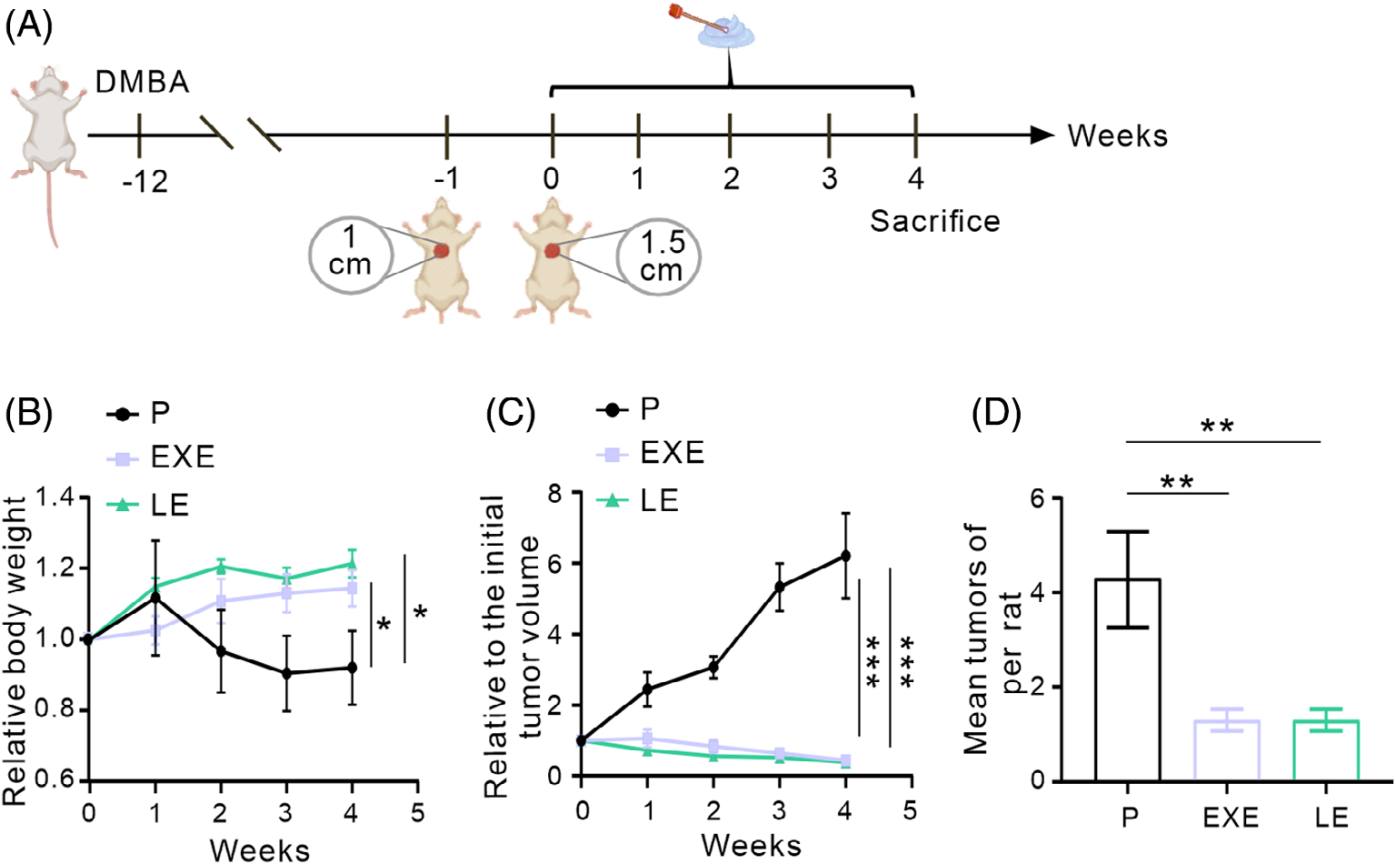
The effects of the creams on breast cancer. (A) Schematic illustration of experimental schedule. (B) Relative body weights of rats in each group. (C) Tumour growth curve. (D) Average total tumour number per rat in each group. Each rat received 125 mg/kg bw/d of placebo cream without drug‐treated animals. EXE, each rat received 125 mg/kg bw/d of EXE cream. LE, each pig received 125 mg/kg bw/d of LE cream.

We evaluated the effectiveness of EXE and LE creams in treating breast cancer in female rats that had been induced with DMBA. The rats with breast cancer underwent ovariectomy, and the tumours were topically treated with EXE or LE creams (Figure 5A). The rats treated with EXE and LE creams regained weight throughout treatment, while the placebo group exhibited gradual weight loss (Figure 5B). The difference in body weights between the experimental and control rats was significant (*p* < 0.01). As expected, the tumours in the control placebo group increased in size. In contrast, treatment with EXE or LE resulted in a growth arrest of the tumours (Figures 5C and S7A,B). At the onset of treatment, the mean tumour count for all groups was 1.1 ± 0.12 tumours per rat. After 28 days of treatment, the tumour counts were 4.3 ± 0.87 for the control group, 1.3 ± 0.23 for the group treated with EXE and 1.1 ± 0.12 for the group treated with LE (Figure 5D). EXE and LE creams significantly reduced the number of new tumours compared to the control group at the end of the treatment period (*p* < 0.01 for both EXE and LE). Further studies found that the application of two separate aromatase inhibitor creams significantly reduced the level of oestrogen E1 in adipose tissue of breast. However, the amount of oestrogen E2 in this tissue remained unchanged, and there was no impact on the levels of both estrogens in the blood (Figure S8). It is worth noting that oestrogen E1 has been associated with the promotion of breast cancer progression.^33^ These findings indicate that both EXE and LE creams, administered at a dose of 125 mg/kg bw/d, can significantly inhibit the growth of breast tumours and control their number by reducing the content of E1. Despite observing damage to animal organs at a dosage of 1440 mg/kg bw/d, we employed a lower dosage of 125 mg/kg bw/d to assess the anti‐tumour effect of LE cream. The reduction in tumours was not accompanied by any significant adverse effects, as evidenced by the absence of abnormal changes in the internal organs of rats.

### 3.8 Estimation of the androgenic activities of the transdermal creams

**FIGURE 6:**
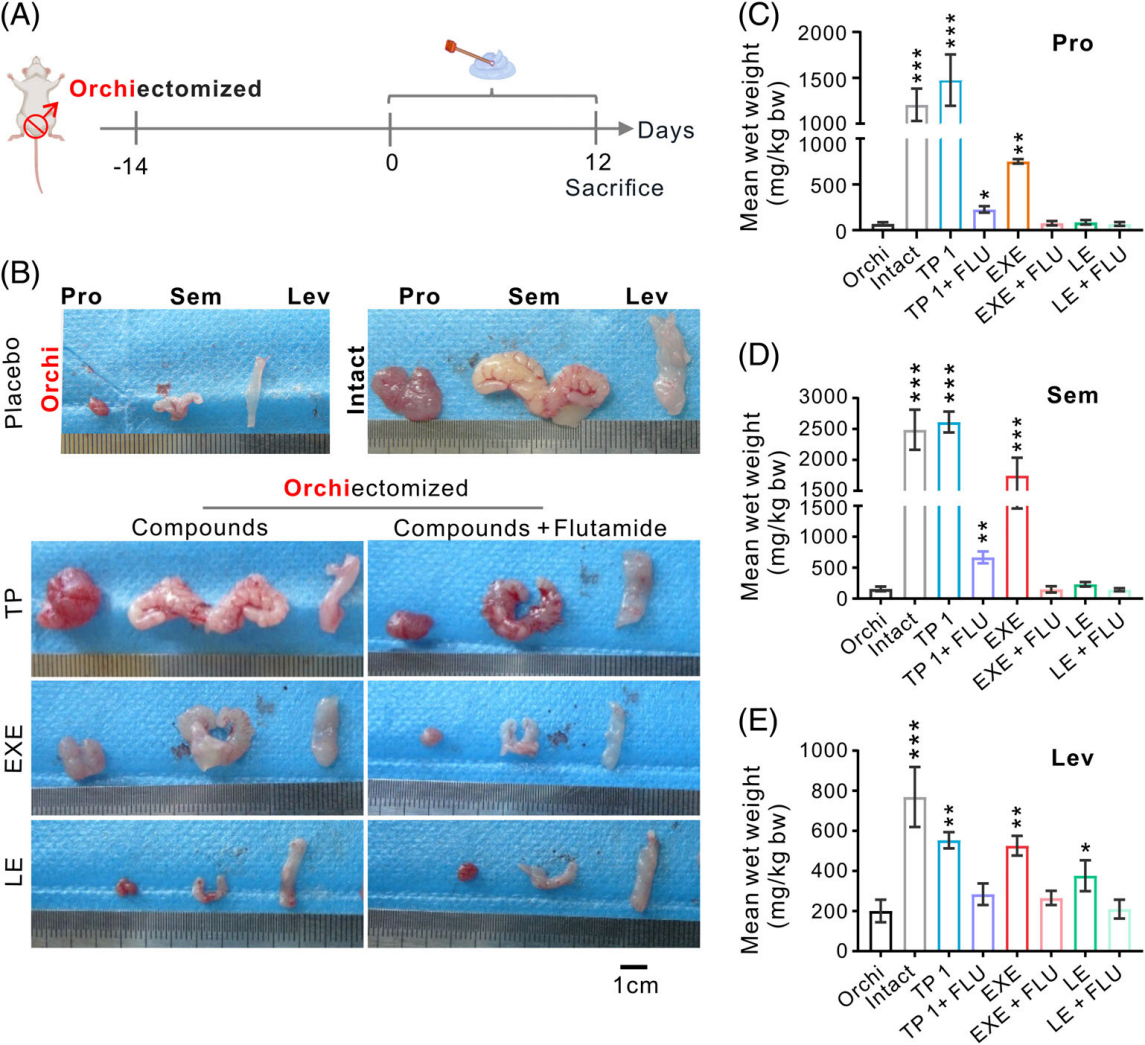
Evaluation of the androgenic activities of the creams. (A) Schematic illustration of experimental schedule. (B) Size and shape of the prostate, seminal vesicle, and levator ani at the end of the treatment period. (C–E) The wet weight of the organs. The numbers at the end of the abbreviations correspond to the dose of the animal in mg/kg bw/d. Flutamide was given by gavage (10 mg/kg bw/d). FLU, flutamide; Lev, levator ani; Orchi, orchiectomized; Pro, prostate; Sem, seminal vesicle; TP, testosterone propionate.

Our previous studies have demonstrated that EXE exerts inhibitory effects on breast cancer progression by activating androgen receptors.^22^ To evaluate the androgenic activity of the creams, we conducted the classic Hershberger assay (Figure 6A). We used testosterone propionate (TP) as a positive control to restore the impact of castration on the prostate, seminal vesicle and levator ani. We also employed the anti‐androgen flutamide (FLU) to prevent this effect (Figure 6B–E). Our results showed that EXE cream at a dose of 125 mg/kg bw/d had a moderate androgenic and anabolic effect, whereas LE and placebo cream at the same dose did not have any such properties. The androgenic effects of EXE cream were eliminated by concomitant oral administration of flutamide (Figure 6B–E). These findings suggested that EXE cream, in addition to its role as an aromatase inhibitor, can also suppress breast cancer growth by activating androgen receptors though the EXE compound itself, rather than other components of the cream. In contrast, LE cream solely functioned as an aromatase inhibitor.

## 4 CONCLUSION

In summary, we developed a nanoemulsion‐based transdermal cream as a carrier for third‐generation aromatase inhibitors EXE and LE. We evaluated their toxicity, anti‐breast cancer effects and androgenic activity in preclinical animal models. When administrated to Bama minipigs at a dosage of 1440 mg/kg bw/d, the EXE cream exhibited no toxicity, while the LE cream caused inflammation, liver function injury and jaundice in animals. Further investigation is necessary to determine the optimal dose for the LE cream. Moreover, administering both creams at a dosage of 125 mg/kg bw/d to rats with breast cancer significantly suppressed tumour growth and reduced the number of tumours. The results of the Hershberger assay revealed that the EXE cream exhibited a moderate androgenic effect, while the LE cream did not. The androgenic activity of the EXE cream partially elucidated its mechanism of action as an anti‐breast cancer agent. Our study suggests that the transdermal cream delivery system suits steroidal (EXE) and non‐steroidal (LE) aromatase inhibitors. Compared to traditional oral dosage forms, direct transdermal local delivery of aromatase inhibitors to breast tumours offers several advantages. It shortens the time and transport pathway for the drug to reach its intended target and bypasses the metabolism and transport processes within the animal system. This, in turn, increases the concentration of the active ingredient in breast tumours. Consequently, the utilization of the drug becomes more efficient and the efficacy and duration of its action are enhanced. In addition, this method of delivery reduces the toxicity associated with drug absorption and metabolism through the gastrointestinal tract. This study lays the foundation for the safe clinical application of transdermal creams containing third‐generation aromatase inhibitors, which offer potential advantages over traditional oral administration.

## AUTHOR CONTRIBUTIONS

**Lanyang Gao**: Conceptualization, methodology, writing–original draft preparation. **Yingying Liu**: Validation, writing–reviewing and editing. **Shiyao Huang**: Data analysis, writing–original draft preparation. **Lei Sun**: Writing–reviewing, software. **Ziming Wu**: Writing–reviewing and editing, software. **Mei Li**: Writing–reviewing. **Chen Shen**: Writing–reviewing. **Youyou Chen**: Methodology. **Ruihao Tan**: Visualization. **Lin Gao**: Writing–reviewing. **Yuji Chen**: Data analysis. **Frank Heinrich Wieland**: Conceptualization, writing–reviewing and supervision. **Yinan Zhang**: Conceptualization, writing–reviewing and editing, supervision. **Yao Luo**: Conceptualization, writing–reviewing and editing, supervision.

## CONFLICT OF INTEREST STATEMENT

The authors declare no conflicts of interest.

## Supporting information


**Data S1.** Supporting Information.

## Data Availability

The data that support the findings of this study are available from the corresponding author upon reasonable request.
