# Supplementary material for: Nanoemulsion‐based transdermal delivery of third‐generation steroidal and non‐steroidal aromatase inhibitors in preclinical models
Source: Cell Prolif. 2024 Sep 29;58(3):e13753. doi: 10.1111/cpr.13753 (PMC11882749; doi:10.1111/cpr.13753)
Supplement: Supplementary file 1 — Data S1. Supporting Information. [file CPR-58-e13753-s001.docx]

Supporting Information

**Nanoemulsion-based transdermal delivery of third-generation steroidal and non-steroidal aromatase inhibitors in preclinical models**

Lanyang Gao^#^, Lin Gao^#^, Shiyao Huang^#^, Lei Sun, Mei Li, Chen Shen, Youyou Chen, Ruihao Tan, Yuji Chen, Chengguo *Z*han, Frank Heinrich Wieland, Yingying Liu^🖂^, Yinan Zhang^🖂^, Yao Luo^🖂^

^#^These authors contributed equally to this work.

^🖂^Correspondence

luoyao@scu.edu.cn

yinan_zhang@tongji.edu.cn

[yangyang21@sjtu.edu.cn](mailto:yangyang21@sjtu.edu.cn)

**This file includes:**

**Supplementary Methods**

**Table S1** Variation in total daily feed intake of Bama minipigs.

**Table S2** Hematology data.

**Table S3** Blood electrolytes and coagulation factors data.

**Table S4** Blood chemistry data for pigs treated by the creams for 28d.

**Table S5** Blood chemistry data during recovery after 28 d of LE cream application.

**Table S6**  Urine analysis for Bama minipigs applied investigational cream for 28 d.

**Table S7** Urine analysis during the recovery period 28 d after application of LE cream.

**Figure S1** The effects of EXE and LE creams on the minipigs’ condition.

**Figure S2** Physiological parameters data of Bama minipigs.

**Figure S3** Blood chemistry data of Bama minipigs.

**Figure S4** Gross anatomical observation of the liver and lung of the minipigs.

**Figure S5** Histopathological examination of minipig organs.

**Figure S6** Histopathological examination of minipig organs.

**Figure S7** The effects of EXE or LE cream on breast cancer.

**Figure S8** Impact of the creams on estrogen content in plasma and adipose tissue of breast.

**SUPPLEMENTARY METHODS**

**Preparation of nanoemulsion-based transdermal creams**

The experimental formulations, Exemestane (EXE) cream and Letrozole (LE) cream were characterized as oil-in-water (O/W) emulsions. These creams comprised 2.5% of the respective active ingredients (Sigma Chemical Co.). A placebo formulation, consisting of purified water without the active compounds, facilitated a comparative analysis between the effects of the active ingredients and those of the placebo in preclinical studies. The patent for these formulations (US 20030092693A1) delineated the composition and manufacturing methods of the cream for its intended use.

**UPLC analysis of compounds in cream chemical components**

The quantification of EXE or LE concentrations in post-administration plasma samples and cream was carried out as reported.^1^ UPLC analysis was performed on a C18 column with dimensions of 2.1 mm inner diameter and 100 mm length, featuring 1.6 μm particle size. A single injection of 5 μL was detected at 278 nm. The isocratic mobile phase consisted of a mixture of water (A) and acetonitrile (B) in a 50 : 50 ratio. The column temperature was maintained at 30 °C, and the flow rate was set at 0.3 mL/min.

**Cream dosage selection**

The initial starting dose for cream was determined based on preclinical studies, according to the recommendations of the FDA. This calculation will consider the body surface area conversion factor (BSA-CF) of 1.1, representing the relationship between minipigs and humans and a 10-fold safety factor. The marketed oral formulations of EXE and LE were administered at a dose level of 25 and 2.5 mg per patient per application, corresponding to a dose of 0.42 and 0.042 mg/kg/application, assuming a mean body weight of 60 kg.^2^ The penetration rate of steroidal compounds through human skin is reported to be approximately 10%.^3^ Given the similar structural characteristics of minipig skin to human skin, it is reasonable to assume that the penetration rate of the compound through the skin of minipigs is also around 10%. We used this information as a reference to determine the daily dose of the active ingredient in EXE cream for minipigs. Translating this to minipigs, the calculated daily dose for the EXE active ingredient in minipigs is 36 mg/kg bw/d, corresponding to 1440 mg/kg bw/d of EXE cream. This calculation is predicated on the assumption that the minipigs' mean body weight (bw) is similar to that of humans, allowing for a proportional adjustment in dosage. Even though LE is a non-steroidal aromatase inhibitor, its safety was assessed using an equivalent amount of EXE cream, specifically 1440 mg/kg bw/d. This dosage represents more than 100 times the clinical dose.

**Animal source and feeding**

Five-month-old female Bama minipigs, deemed healthy with a weight range of 14 to 18 kg, were procured from Chengdu Dashuo Experimental Animal Co. LTD (Certificate of Conformity No.: SCXK(111)20113-24). These pigs were individually housed in cages under standard conditions, with room temperature between 20 ℃ to 25 ℃, relative humidity set at 60% to 80%, and a light-dark cycle of 12 hrs each. Daily care involved the cleaning and sanitizing of cages, with a regular and quantified provision of pig feed and free access to drinking water. The animals underwent a 2-week acclimatization period to adapt to the laboratory conditions before the initiation of the experiment.

Female Sprague-Dawley (SD) or male Wistar rats were supplied by Chengdu Dashuo Experimental Animal Co. Ltd. Rats were housed at five per cage under a regimen of 14 hrs of light and 10 hrs of darkness (lights on at 05:00 a.m.). Animals received rat chow and water ad libitum.

The animal experiments were performed under the Guideline for Care and Use of Experimental Animals and approved by the Institutional Review Board (or Ethics Committee) of The Affiliated Hospital of Southwest Medical University, Southwest Medical University (protocol code 201903-37 and date of approval 2019-03-05).

**Application method of the creams in toxicity assessment experiment**

Before administration, the dorsal hair on both sides of the Bama minipigs was removed using an electric hair remover, resulting in an approximately 25 cm × 25 cm hair removal area. The specific experimental area designated for cream application measured 20 cm × 20 cm. The cream was uniformly applied to this area, ensuring even coverage. Subsequently, the treated area was covered with sulfuric acid paper and securely fastened using special fixation tape. After a 24-hour period, any remaining cream was carefully removed using warm, soapy water. This method was employed to guarantee a consistent and uniform application of the cream across the designated treatment area. Upon the conclusion of the experimental period on day 28, animals deemed to be in good condition underwent necropsy and macroscopic examination. However, those exhibiting poor conditions were allowed an additional 3-week recovery period.

**Clinical observation in toxicity assessment experiment**

Throughout the 28-day study period, the Bama minipigs underwent meticulous monitoring to assess potential adverse effects from exposure to the two creams. Weekly measurements of body weight and temperature were taken. Additionally, the animals' body temperature was evaluated at specific time points, including 0, 4, 8, 12, 24, 48 and 96 hrs post-treatment with EXE or LE cream. Food consumption per pig was documented once a week, following the methodology outlined in relevant literature.^4^ Moreover, the animals’ responses to the administered drugs were meticulously observed within a 4-hour post-administration window. A comprehensive functional observational battery was systematically conducted on all subjects to assess various parameters, including excitability, autonomic function, gait, sensorimotor coordination, reactivity, sensitivity and the manifestation of any abnormal clinical signs. Upon the culmination of the study period, the animals were euthanized, and thorough hematological and urine analyses were conducted to evaluate the potential toxicity of the administered creams. Histological analyses were also undertaken to scrutinize the effects of the creams on the organs of the Bama minipigs, providing a detailed understanding of the physiological impact of the experimental treatments.

**Mammary tumor model**

Six-week-old female SD rats weighing approximately 150 g were utilized. The induction of mammary carcinomas was carried out through two oral administrations of DMBA (Sigma Chemical Co.) at a cumulative dose of 20 mg/rat, administered at 6 and 7 weeks of age. Starting 40 days post-DMBA treatment, animals underwent weekly palpation examinations. When at least one tumor measuring 1 cm in diameter was found, the rats were ovariectomized. Then, rats exhibiting at least one tumor with a diameter of 1.5 cm or larger were sequentially assigned to experimental groups, each comprising 10 rats. Weekly measurements of the rats’ body weights were recorded. Animals without tumors were excluded from the study 150 days post-DMBA treatment. The two perpendicular tumor axes were measured with calipers twice a week. Tumor volume was calculated by the formula d^2^ × D/2, where d is the minimal and D is the maximal diameter.

**Routine uri****nalysis**

The study systematically collected urine samples from all animals using metabolic cages for approximately 16 hrs overnight. This collection occurred during two distinct periods: firstly, during the pre-study phase (overnight from study day -5 to -7) and secondly, after the treatment period (overnight from study day 6, day 13, day 20 and day 27). Importantly, these collections were conducted while the animals were fasting. The purpose of these urine samples was to conduct a thorough analysis to evaluate the potential toxicity of the administered creams on the renal function of the animals.

The urine analysis of all animals was performed in a laboratory using a urine analyzer (MI-921D, Wuhan Changfeng Medical Device Co., LTD, China). Various parameters, including urine specific gravity (SG), pH, white blood cell count (WBC), nitrite (NIT), urine protein (PRO), glucose (GLU), ketones (KET), urobilinogen (URO), bilirubin (BIL) and urine occult blood (BLD), were systematically evaluated. These assessments were conducted every week throughout the test period, enabling continuous monitoring for any signs of potential toxicity. The urine analysis was performed according to standard procedures to ensure the accuracy and consistency of the results.

**Hematology and serum biochemistry analysis**

Blood samples were procured from each animal on the following occasions: before the commencement of the study and subsequently once every week during the study period. In preparation for blood collection, the animals underwent a fasting period without access to water beginning from the afternoon of the day preceding the scheduled blood collection. Blood was collected from the anterior vena cava without the use of anesthesia, adhering to established procedures.

Hematological indexes: 1 mL of whole blood was anticoagulated with EDTA and the red blood cell count (RBC), WBC, hemoglobin (HGB), red blood cell ratio (HCT), mean red blood cell volume (MCV) and mean red blood cell hemoglobin (MCH), mean hemoglobin concentration (MCHC), red blood cell volume distribution width (RDW), platelet count (PLT), mean platelet volume (MPV), lymphocyte count (LY), Intermediate cell absolute value (MID) and other indicators were measured by automatic hemocytometer (TEK-ⅡMINI, Jiangxi Tecon Technology Co., China).

Detection of serum biochemical indexes: 3 mL of non-anticoagulated blood was centrifuged, and the serum was analyzed by an automatic biochemical analyzer (TC6010L, Jiangxi Tecon Technology Co., China) for alanine aminotransferase (ALT), aspartate aminotransferase (AST), alkaline phosphatase (ALP), total protein (TP), albumin (ALB), creatine kinase (CK), creatinine (CRE), urea (BUN), total cholsolid alcohol (TC), glucose (GLU), triglyceride (TG), potassium (K^+^), sodium (Na^+^), chlorine (Cl^-^), calcium (Ca^2+^) and other biochemical indexes.

Blood coagulation indexes: 1 mL of whole blood was anticoagulated with sodium citrate, the plasma was separated by centrifugation, and the two indexes of prothrombin time (PT) and activated partial thromboplastin time (APTT) were measured by Semi-automatic hemagg-lutinometer (PUN-2048B, Beijing Plantronics Technology Co., China).

**REFERENCES**

1. Huang S, Wu Z, Chen Y, et al. Dermal repeated dose toxicity study of the anti-breast cancer drug Formestane cream in Bama minipig. *Food Chem Toxicol.* 2023;178:113927.

2. Dowsett M, Goss PE, Powles TJ, et al. Use of the aromatase inhibitor 4-hydroxyandrostenedione in postmenopausal breast cancer: optimization of therapeutic dose and route. *Cancer Res.* 1987;47(7):1957-1961.

3. Feldmann RJ, Maibach HI. Percutaneous penetration of steroids in man. *J Invest Dermatol.* 1969;52(1):89-94.

4. Schmitt D, Tran N, Peach J, Bauter M, Marone P. Toxicologic evaluation of DHA-rich algal oil: Genotoxicity, acute and subchronic toxicity in rats. *Food Chem Toxicol.* 2012;50(10):3567-3576.

**Table S1** Variation in total daily feed intake of Bama minipigs.

| Time  [weeks] | Total daily feed intake (± SD, g)^a)^ | | | |
| --- | --- | --- | --- | --- |
|  | N | P | EXE | LE |
| -1 | 3746 ± 356 | 3842 ± 279 | 3766 ± 399 | 3620 ± 243 |
| 0 | 3766 ± 296 | 3697 ± 243 | 3801 ± 394 | 3905 ± 255 |
| 1 | 3710 ± 270 | 3585 ± 116 | 3611 ± 119 | 3668 ± 368 |
| 2 | 3550 ± 199 | 3614 ± 418 | 3673 ± 223 | 3720 ± 233 |
| 3 | 3835 ± 358 | 3709 ± 164 | 3680 ± 381 | 3651 ± 178 |
| 4 | 3608 ± 410 | 3668 ± 115 | 3739 ± 196 | 2172 ± 289 *^b)^ |
| 5 | 3723 ± 310 | -^c)^ | - | 2239 ± 192 * |
| 6 | 3615 ± 280 | - | - | 2966 ± 170 * |
| 7 | 3684 ± 419 | - | - | 3615 ± 216 |

^a)^ Data of each group was presented as mean ± SD (*n* = 4). ^b)^ **p* ≤ 0.05 *vs.* Normal. ^C)^ “-” means no data.

**Table S2** Hematology data.

| Days | Parameters^a)^ | N | P | EXE | LE |
| --- | --- | --- | --- | --- | --- |
| 0 | RBC [10E12/L] | 6.72 ± 0.19 | 6.29 ± 0.36 | 6.7 ± 0.8 | 6.08 ± 0.36 |
|  | Hgb [g/L] | 114 ± 4.83 | 111.5 ± 5.92 | 112.5 ± 14.43 | 105.75 ± 8.14 |
|  | MCHC [g/L] | 366.5 ± 8.66 | 370.25 ± 8.26 | 364.75 ± 2.87 | 369.25 ± 15.46 |
|  | PLT [10E9/L] | 816.75 ± 300.13 | 621 ± 286.36 | 651 ± 157.13 | 494.25 ± 90.16 |
|  | WBC [10E9/L] | 15.3 ± 4.01 | 19.43 ± 6.31 | 25.08 ± 12.61 | 15 ± 3.20 |
|  | LY [10E9/L] | 7.85 ± 0.64 | 9.65 ± 3.57 | 11.63 ± 6.37 | 6.38 ± 2.39 |
|  | MID [10E9/L] | 0.95 ± 0.44 | 1.43 ± 0.33 | 1.58 ± 0.62 | 1.15 ± 0.42 |
| 28 | RBC [10E12/L] | 7.04 ± 0.32 | 6.61 ± 0.3 | 7.02 ± 0.16 | 5.03 ± 2.98 |
|  | Hgb [g/L] | 113.5 ± 2.89 | 112.75 ± 8.02 | 116 ± 9.06 | 120.5 ± 6.66 |
|  | MCHC [g/L] | 352 ± 5.29 | 350.25 ± 14.34 | 350 ± 10.23 | 316.25 ± 58.82 |
|  | PLT [10E9/L] | 833.75 ± 291.65 | 504.5 ± 152.73 | 831.5 ± 389.82 | 567 ± 285.67 |
|  | WBC [10E9/L] | 18.83 ± 3.61 | 14.2 ± 1.37 | 28.25 ± 13.8 | 43.03 ± 6.99 **↑ ^c)^ |
|  | LY [10E9/L] | 9.9 ± 3.88 | 6.23 ± 0.85 | 17.75 ± 11.48 | 21.83 ± 10.68 |
|  | MID [10E9/L] | 1.35 ± 0.24 | 1.1 ± 0.16 | 1.73 ± 0.34 | 1.88 ± 0.26 |
| 35 | RBC [10E12/L] | 6.58 ± 0.29 | - ^b)^ | - | 7.56 ± 0.05 |
|  | Hgb [g/L] | 116 ± 2.83 | - | - | 127.5 ± 6.36 *↑ |
|  | MCHC [g/L] | 349 ± 15.56 | - | - | 337.5 ± 0.71 |
|  | PLT [10E9/L] | 444 ± 63.64 | - | - | 723.5 ± 30.41 |
|  | WBC [10E9/L] | 13.75 ± 3.32 | - | - | 61.1 ± 1.56 ***↑ |
|  | LY [10E9/L] | 5.55 ± 1.34 | - | - | 42.1 ± 5.23 ***↑ |
|  | MID [10E9/L] | 1.2 ± 0.28 | - | - | 1.9 ± 0.28 *↑ |
| 49 | RBC [10E12/L] | 6.88 ± 0.69 | - | - | 6.64 ± 0.02 |
|  | Hgb [g/L] | 112 ± 4.24 | - | - | 124.5 ± 9.19 |
|  | MCHC [g/L] | 360.5 ± 0.71 | - | - | 379.5 ± 10.61 |
|  | PLT [10E9/L] | 510.5 ± 86.97 | - | - | 633 ± 26.87 |
|  | WBC [10E9/L] | 17.7 ± 3.25 | - | - | 53.25 ± 5.44 ***↑ |
|  | LY [10E9/L] | 7.3 ± 2.4 | - | - | 36.6 ± 6.51***↑ |
|  | MID [10E9/L] | 1.2 ± 0.28 | - | - | 1.95 ± 0.07 *↑ |

^a)^ Values are mean ± SME for groups of 4 minipigs. ^b)^  “-” mean no data. ^c)^ Asterisks and arrows are used to indicate significant differences and increasing trends in the LE group compared to the normal group.

**Table S3** Blood electrolytes and coagulation factors data.

| Days | Parameters^a)^ | N | P | EXE | LE |
| --- | --- | --- | --- | --- | --- |
| 0 | K^+^ [mmol/L] | 4.46 ± 0.59 | 4.8 ± 0.21 | 4.58 ± 0.44 | 4.4 ± 0.23 |
|  | Na^+^ [mmol/L] | 145.78 ± 1.1 | 145.98 ± 2.36 | 144.33 ± 1.61 | 143.63 ± 2.65 |
|  | Cl^-^ [mmol/L] | 101.68 ± 2.51 | 99.68 ± 3.73 | 98.45 ± 1.28 | 98.48 ± 2.72 |
|  | Ca^2+^ [mmol/L] | 2.13 ± 0.05 | 2.09 ± 0.12 | 2.09 ± 0.09 | 2.12 ± 0.04 |
|  | PT [S] | 11.35 ± 1.28 | 11.5 ± 2.75 | 11.58 ± 2.76 | 11.98 ± 1.25 |
|  | APTT [S] | 18.13 ± 1.93 | 17.45 ± 1.84 | 17.05 ± 0.9 | 19.35 ± 1.76 |
| 28 | K^+^ [mmol/L] | 4.26 ± 0.34 | 4.5 ± 0.19 | 4.31 ± 0.16 | 4.44 ± 0.22 |
|  | Na^+^ [mmol/L] | 142.58 ± 0.79 | 145.08 ± 3.48 | 141.98 ± 1.16 | 143.03 ± 1.72 |
|  | Cl^-^ [mmol/L] | 99.05 ± 1.12 | 100.1 ± 5.57 | 98.6 ± 1.55 | 99.35 ± 1.17 |
|  | Ca^2+^ [mmol/L] | 2.41 ± 0.05 | 2.39 ± 0.18 | 2.31 ± 0.11 | 2.31 ± 0.12 |
|  | PT [S] | 9.45 ± 0.29 | 9.03 ± 0.51 | 9.95 ± 0.68 | 9.93 ± 0.33 |
|  | APTT [S] | 17.68 ± 0.79 | 17.78 ± 0.67 | 17.05 ± 1.32 | 18.45 ± 0.95 |
| 35 | K^+^ [mmol/L] | 4.23 ± 0.17 | -^b)^ | - | 5.27 ± 0.3 |
|  | Na^+^ [mmol/L] | 137.15 ± 1.06 | - | - | 133.8 ± 0.28 |
|  | Cl^-^ [mmol/L] | 102.4 ± 1.41 | - | - | 101.2 ± 0 |
|  | Ca^2+^ [mmol/L] | 2.52 ± 0.02 | - | - | 2.73 ± 0.04 |
|  | PT [S] | 9.5 ± 0.85 | - | - | 9.7 ± 0.14 |
|  | APTT [S] | 20.95 ± 0.64 | - | - | 18.05 ± 2.05 |
| 49 | K^+^ [mmol/L] | 4.6 ± 0.08 | - | - | 4.98 ± 0.37 |
|  | Na^+^ [mmol/L] | 140.05 ± 1.06 | - | - | 139.7 ± 4.10 |
|  | Cl^-^ [mmol/L] | 101 ± 0.42 | - | - | 101.55 ± 1.48 |
|  | Ca^2+^ [mmol/L] | 2.68 ± 0.06 | - | - | 2.85 ± 0.07 |
|  | PT [S] | 9.7 ± 0.85 | - | - | 9.45 ± 0.07 |
|  | APTT [S] | 20.55 ± 1.06 | - | - | 20 ± 0.57 |

^a)^ Values are mean ± SD for groups of 4 minipigs. ^b)^  “-” mean no data.

**Table S4** Blood chemistry data for pigs treated by the creams for 28d.

| Days | Parameters^a)^ | N | P | EXE | LE |
| --- | --- | --- | --- | --- | --- |
| 0 | ALT [U/L] | 40.03 ± 15.15 | 44.18 ± 7.36 | 53.8 ± 9.27 | 53.58 ± 12.03 |
|  | AST [U/L] | 107.8 ± 126.32 | 22.33 ± 8.28 | 79.9 ± 28.71 | 79.6 ± 49.61 |
|  | TP [g/L] | 69.35 ± 6.03 | 63.93 ± 11.51 | 68.43 ± 3.19 | 72.5 ± 0.95 |
|  | ALB [g/L] | 30.23 ± 1.72 | 28.38 ± 3.94 | 32.23 ± 2.17 | 30.38 ± 1.19 |
|  | GLO [g/L] | 40.15 ± 4.29 | 35.15 ± 8.68 | 36.15 ± 1.53 | 42 ± 1.14 |
|  | A/G | 0.78 ± 0.1 | 0.84 ± 0.17 | 0.81 ± 0.06 | 0.68 ± 0.04 |
|  | TB [µmol/L] | 13.03 ± 1.66 | 13.63 ± 1.74 | 12.75 ± 1.93 | 13.35 ± 1.51 |
|  | BUN [mmol/L] | 2.17 ± 0.5 | 2.55 ± 0.37 | 2.6 ± 0.55 | 2.55 ± 0.41 |
|  | CRE [µmol/L] | 89.45 ± 11.03 | 78.85 ± 10.33 | 88.6 ± 10.58 | 72.78 ± 5.19 |
|  | GLU [mmol/L] | 3.91 ± 0.95 | 3.92 ± 1.08 | 3.73 ± 0.96 | 3.65 ± 0.43 |
|  | TC [mmol/L] | 1.89 ± 0.41 | 1.95 ± 0.46 | 1.69 ± 0.21 | 1.61 ± 0.25 |
|  | TG [mmol/L] | 0.28 ± 0.06 | 0.46 ± 0.23 | 0.27 ± 0.08 | 0.26 ± 0.12 |
|  | CK [U/L] | 452.03 ± 265.75 | 353.65 ± 144.06 | 452.88 ± 270.23 | 207.83 ± 31.16 |
| 28 | ALT [U/L] | 48.65 ± 14.59 | 64.43 ± 23.98 | 164.7 ± 125.8 | 346 ± 3 42.14 |
|  | AST [U/L] | 63.48 ± 11.48 | 66.7 ± 50.72 | 219 ± 149.74 | 504.85 ± 281.15 **↑ ^b)^ |
|  | TP [g/L] | 73.03 ± 4.17 | 68.93 ± 7.37 | 69.8 ± 4.52 | 77.63 ± 4.63 |
|  | ALB [g/L] | 32.78 ± 2.7 | 31.98 ± 2.89 | 35.63 ± 1.62 | 34.13 ± 1.96 |
|  | GLO [g/L] | 40.28 ± 4.13 | 37.18 ± 6.19 | 34.18 ± 3.4 | 43.5 ± 5.27 |
|  | A/G | 0.81 ± 0.09 | 0.88 ± 0.15 | 1.05 ± 0.08 | 0.8 ± 0.12 |
|  | TB [µmol/L] | 1.23 ± 0.34 | 1.55 ± 0.17 | 2.4 ± 3.67 | 36.95 ± 39.66 |
|  | BUN [mmol/L] | 2.21 ± 0.5 | 2.26 ± 0.34 | 3.75 ± 0.62 | 3.82 ± 0.95 |
|  | CRE [µmol/L] | 79.9 ± 7.21 | 70.38 ± 10.34 | 77.3 ± 8.48 | 70.83 ± 13.84 |
|  | GLU [mmol/L] | 3.38 ± 0.92 | 3.43 ± 0.41 | 3.12 ± 0.18 | 3.08 ± 0.5 |
|  | TC [mmol/L] | 2.23 ± 0.51 | 2.25 ± 0.07 | 2.71 ± 0.54 | 2.39 ± 0.81 |
|  | TG [mmol/L] | 0.29 ± 0.12 | 0.32 ± 0.12 | 0.29 ± 0.08 | 0.47 ± 0.32 |
|  | CK [U/L] | 376.93 ± 136.12 | 444.18 ± 178.4 | 509.75 ± 321.97 | 423.68 ± 103.58 |

^a)^ Values are mean ± SD for groups of 4 minipigs. ^b)^ Asterisks and arrows are used to indicate significant differences and increasing trends in the LE group compared to the normal group.

**Table S5** Blood chemistry data during recovery after 28 d of LE cream application.

| Parameters^a)^ | 35 d | |  | 49 d | |
| --- | --- | --- | --- | --- | --- |
|  | N | LE |  | N | LE |
| ALT [U/L] | 54.5 ± 10.47 | 279.2 ± 32.39 |  | 65.3 ± 15.98 | 232.1 ± 34.22 |
| AST [U/L] | 88.65 ± 98.08 | 698.8 ± 160.6 ***↑ ^b)^ |  | 74.5 ± 44.55 | 499.15 ± 137.3 ***↑ |
| TP [g/L] | 69.9 ± 7.21 | 71.95 ± 3.04 |  | 67.1 ± 0.85 | 66.35 ± 1.34 |
| ALB [g/L] | 33.8 ± 2.12 | 34.5 ± 1.7 |  | 35.2 ± 6.51 | 32.6 ± 2.97 |
| GLO [g/L] | 36.1 ± 9.33 | 37.45 ± 4.74 |  | 36.75 ± 0.35 | 33.75 ± 1.63 |
| A/G | 0.98 ± 0.31 | 0.94 ± 0.16 |  | 0.89 ± 0.04 | 0.97 ± 0.13 |
| TB [µmol/L] | 9.95 ± 0.92 | 21 ± 7.35 |  | 11.45 ± 0.92 | 25.3 ± 10.32 |
| BUN [mmol/L] | 3.93 ± 0.86 | 4.92 ± 0.58 |  | 2.86 ± 0.2 | 4.24 ± 0.18 |
| CRE [µmol/L] | 67.8 ± 3.39 | 63 ± 3.39 |  | 97.8 ± 1.13 | 68.4 ± 2.55 |
| GLU [mmol/L] | 3.64 ± 0.03 | 2.47 ± 0.02 |  | 3.27 ± 0.72 | 2.38 ± 0.23 |
| TC [mmol/L] | 2.63 ± 0.53 | 2.51 ± 0.18 |  | 2.6 ± 0.24 | 2.61 ± 0.6 |
| TG [mmol/L] | 0.6 ± 0.26 | 0.34 ± 0.01 |  | 0.29 ± 0.05 | 0.29 ± 0.01 |
| CK [U/L] | 322.1 ± 54.87 | 537.3 ± 58.83 |  | 349.2 ± 26.3 | 317.75 ± 73.75 |

^a)^ Values are mean ± SD for groups of 3 minipigs. ^b)^ Asterisks and arrows are used to indicate significant differences and increasing trends in the LE group compared to the normal group.

**Table S6** Urine analysis for Bama minipigs applied investigational cream for 28 d.

| Days | Parameters | N | P | EXE | LE |
| --- | --- | --- | --- | --- | --- |
| 0 | GLU [mmol/L] | -^b)^ | - | - | - |
|  | WBC [/HP] | - | - | - | - |
|  | NIT | - | - | - | - |
|  | PRO [g/L] | - | - | - | - |
|  | BLD [Ery/µL] | - | - | - | - |
|  | KET [mmol/L] | - | - | - | - |
|  | BIL [µmol/L] | - | - | - | - |
|  | URO [µmol/L] | norm | norm | norm | norm |
| 7 | GLU [mmol/L] | - | - | - | - |
|  | WBC [/HP] | - | - | - | - |
|  | NIT | 1 + (1)^a)^ | 1 + (2) | 1 + (1) | 1 + (3)^c)^ |
|  | PRO [g/L] | 1 + (1) | - | - | +- (1) ^d)^ |
|  | BLD [Ery/µL] | - | - | 2 + (1) | 1 + (1) |
|  | KET [mmol/L] | - | - | - | - |
|  | BIL [µmol/L] | - | - | - | - |
|  | URO [µmol/L] | norm | norm | norm | norm |
| 14 | GLU [mmol/L] | - | - | - | - |
|  | WBC [/HP] | - | - | - | - |
|  | NIT | - | - | - | - |
|  | PRO [g/L] | 1 + (1) | 1 + (1) | - | 2 + (1) |
|  | BLD [Ery/µL] | - | - | - | - |
|  | KET [mmol/L] | - | - | - | - |
|  | BIL [µmol/L] | - | - | - | 3 + (1) |
|  | URO [µmol/L] | norm | norm | norm | 1 + (1), 2 + (1) |
| 21 | GLU [mmol/L] | - | - | - | - |
|  | WBC [/HP] | - | - | - | - |
|  | NIT | - | 1 + (1) | - | - |
|  | PRO [g/L] | - | - | - | - |
|  | BLD [Ery/µL] | - | 1 + (1) | - | - |
|  | KET [mmol/L] | - | - | - | - |
|  | BIL [µmol/L] | - | - | - | 1 + (1) |
|  | URO [µmol/L] | norm | norm | norm | 1 + (1) |
| 28 | GLU [mmol/L] | - | - | - | - |
|  | WBC [/HP] | - | - | - | - |
|  | NIT | 1 + (1) | 1 + (1) | - | - |
|  | PRO [g/L] | - | - | - | 1 + (1) |
|  | BLD [Ery/µL] | - | - | - | - |
|  | KET [mmol/L] | - | - | - | - |
|  | BIL [µmol/L] | - | - | - | 1 + (1) |
|  | URO [µmol/L] | norm | norm | norm | 1 + (1) |

^a)^ The values of the brackets mean the number of minipigs. ^b)^ “-” indicates a negative degree. ^c, d)^ “+-” and “+” indicate a positive degree.

**Table S7** Urine analysis during the recovery period 28 d after application of LE cream.

| Days | Parameters | N | LE |
| --- | --- | --- | --- |
| 35 | GLU [mmol/L] | - | - |
|  | WBC [/HP] | - | - |
|  | NIT | 1 + (1)^a)^ | 1 + (2)^c)^ |
|  | PRO [g/L] | -^b)^ | - |
|  | BLD [Ery/µL] | - | - |
|  | KET [mmol/L] | - | - |
|  | BIL [µmol/L] | - | - |
|  | URO [µmol/L] | norm | norm |
| 42 | GLU [mmol/L] | - | - |
|  | WBC [/HP] | - | - |
|  | NIT | - | 1 + (1) |
|  | PRO [g/L] | - | - |
|  | BLD [Ery/µL] | - | - |
|  | KET [mmol/L] | - | - |
|  | BIL [µmol/L] | - | - |
|  | URO [µmol/L] | norm | norm |
| 49 | GLU [mmol/L] | - | - |
|  | WBC [/HP] | - | - |
|  | NIT | 1 + (1) | - |
|  | PRO [g/L] | - | +- (1)^d)^ |
|  | BLD [Ery/µL] | - | - |
|  | KET [mmol/L] | - | - |
|  | BIL [µmol/L] | - | - |
|  | URO [µmol/L] | norm | norm |

^a)^ The values of the brackets mean the number of minipigs. ^b)^ “-” indicates a negative degree. ^c, d)^ “+-” and “+” indicate a positive degree.


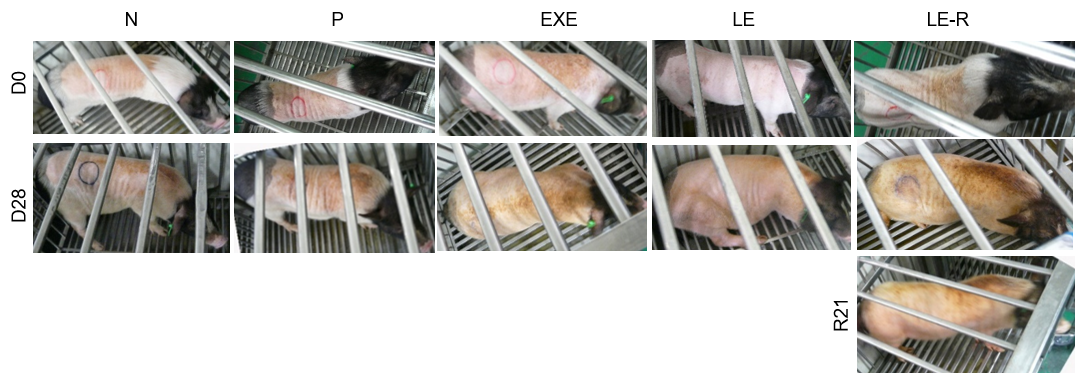


**Figure S1** The effects of EXE and LE creams on the minipigs’ condition. N, Normal, pigs without any treatment. P, each pig received 1440 mg/kg bw/d of placebo cream. EXE, each pig received 1440 mg/kg bw/d of EXE cream. LE, each pig received 1440 mg/kg bw/d of LE cream. LE-R, recovery continued for an additional 3 weeks after day 28 in the minipigs from the LE group. R21, recovery continued for an additional 21 days after day 28 in the minipigs from the LE group.


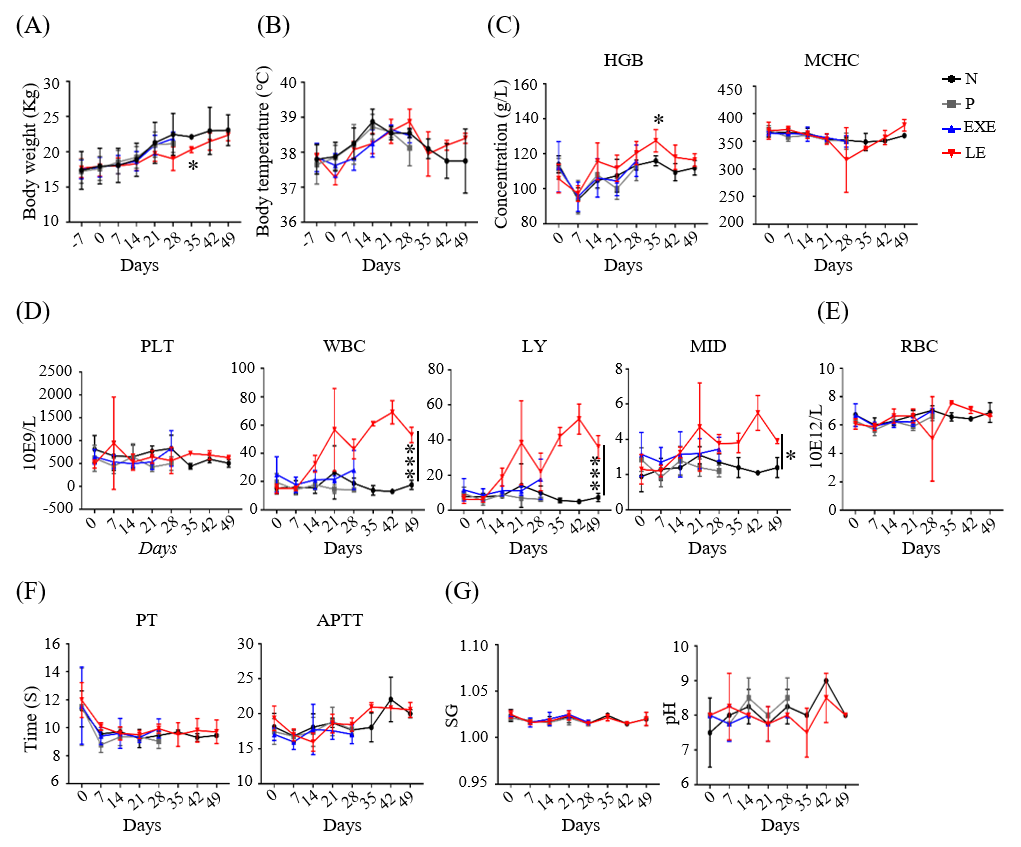


**Figure S2** Physiological parameters data of Bama minipigs. (A, B) Mean body weight and body temperature data. (C-E) The hematological results for the animals. (F) The data of coagulation factors in the blood. (G) The values of urine specific gravity and pH. Values are means ± SME (*n* = 4), and no statistically significant differences were observed between groups at the same time.


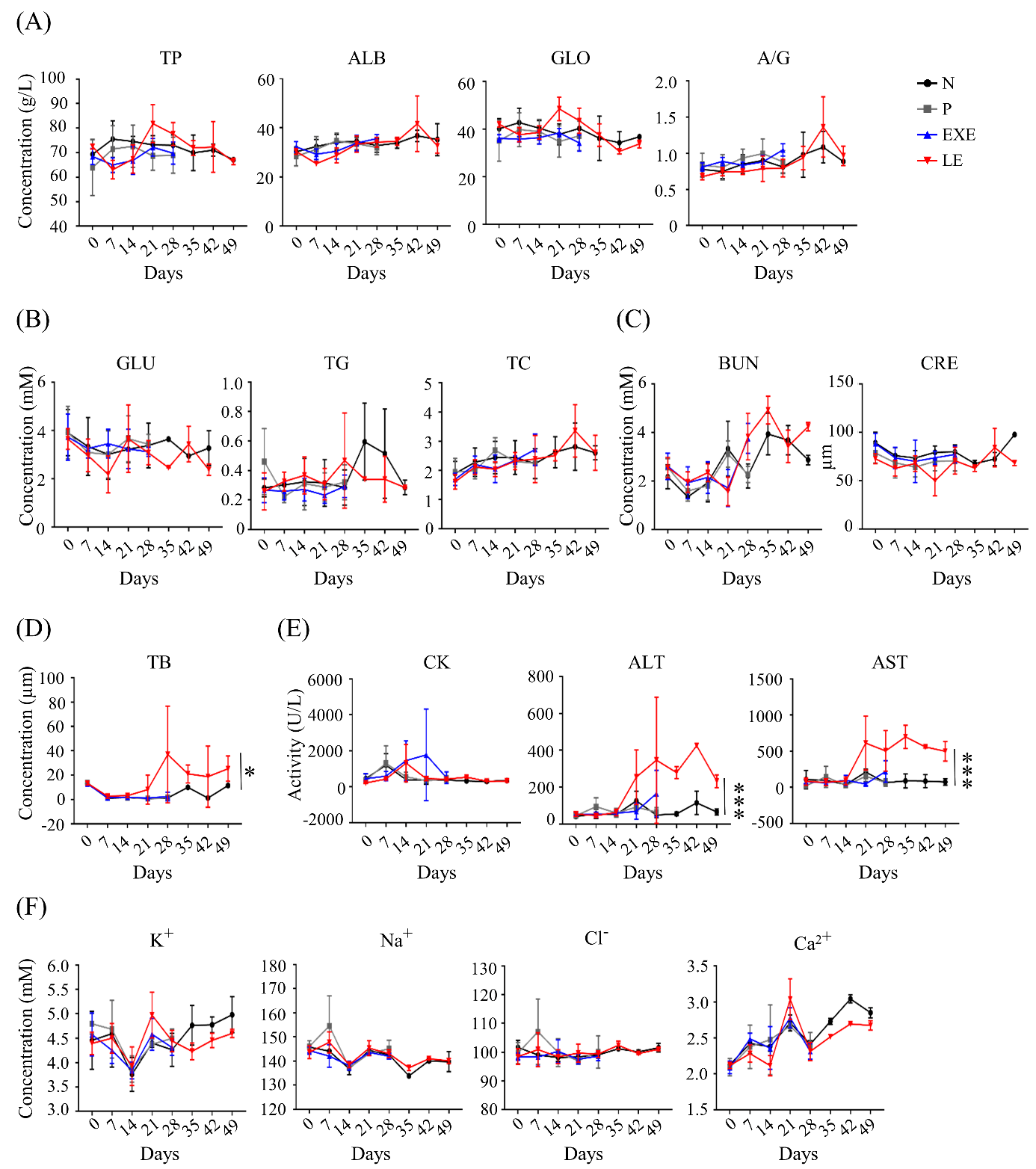


**Figure S3** Blood chemistry data of Bama minipigs. (A) The values of TP, ALB, GLO, and A/G. (B) The levels of GLU, TG, and TC. (C) The levels of BUN and CRE. (D) The concentration of TB in serum. (E) Enzyme activities of CK, ALT, and AST. (F) Blood electrolytes data.

**
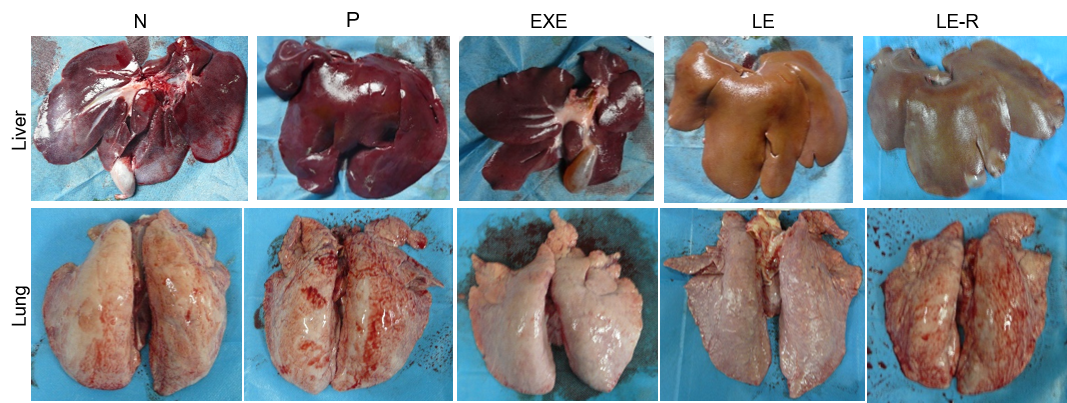
**

**Figure S4** Gross anatomical observation of the liver and lung of the minipigs. N, normal, pigs without any treatment. P, each pig received 1440 mg/kg bw/d of placebo cream. EXE, each pig received 1440 mg/kg bw/d of EXE cream. LE, each pig received 1440 mg/kg bw/d of LE cream. LE-R, recovery continued for an additional 3 weeks after day 28 in the minipigs from the LE group.


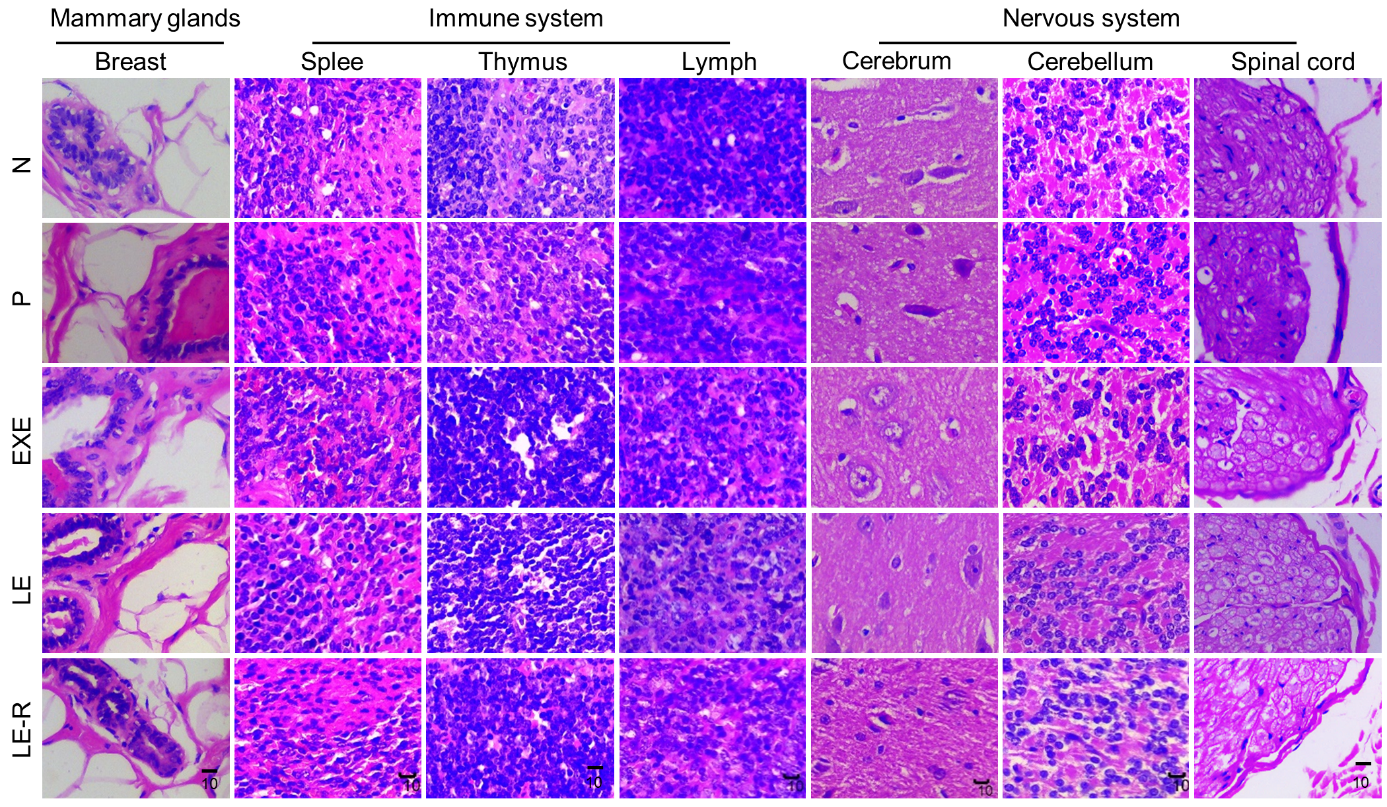


**Figure S5** Histopathological examination of minipig organs. Sections observed at 400 × magnification showed histological structures for the mammary system (breast), immune system (spleen, thymus, and lymph), and nervous system (cerebrum, cerebellum, and spinal cord). Scale bar: 10 μm.


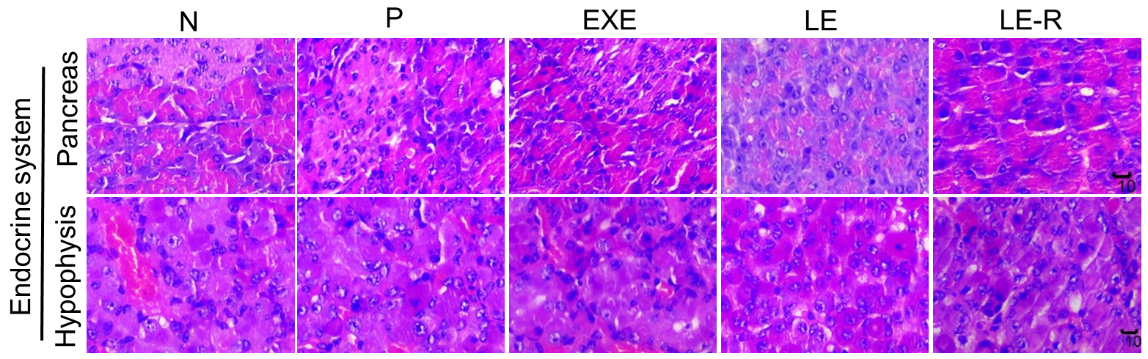


**Figure S6** Histopathological examination of minipig organs. Sections observed at 400 × magnification showed histological structures for the endocrine system (hypophysis, pancreas). Scale bar: 10 μm.


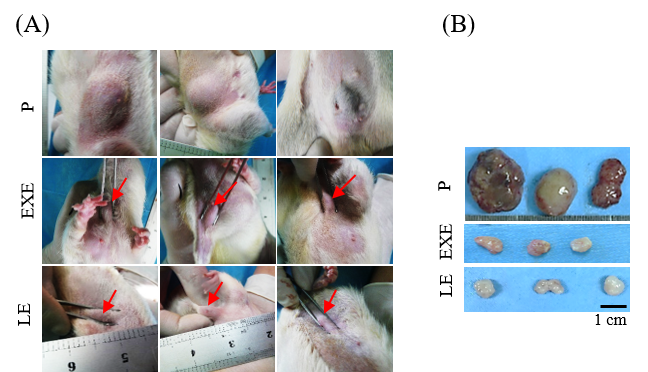


**Figure S7** The effects of EXE or LE cream on breast cancer. (A, B) Representative images of the tumors. P, Placebo cream. EXE, Exemestane cream. LE, Letrozole cream.


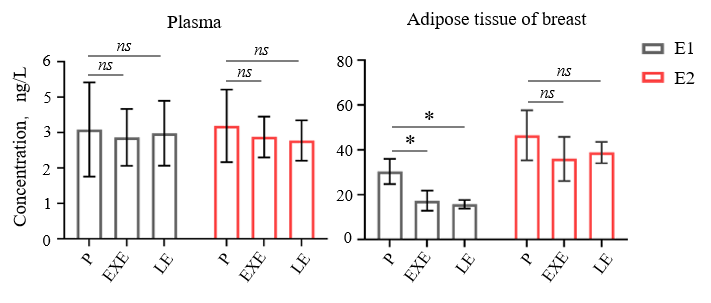


**Figure S8** Impact of the creams on estrogen content in plasma and adipose tissue of breast. P, Placebo cream. EXE, Exemestane cream. LE, Letrozole cream. E1, Oestrone. E2, Estradiol. A *p*-value less than 0.05 is considered statistically significant, while *ns* indicates nonsignificance. Significance levels were denoted by asterisks (**p* < 0.05, ***p* < 0.01, ****p* < 0.001).
